# Right Ventricular Clot in Transit in a Patient with Coronavirus Disease 2019 (COVID-19) Pneumonia Successfully Treated with Thrombolytics

**DOI:** 10.1155/2022/3483605

**Published:** 2022-07-05

**Authors:** Yaman Alali, Ian Jackson, Abedel Rahman Anani, Marisa Varghese, Muhammad Ebrahim Khan, Suchitra Pilli

**Affiliations:** ^1^Department of Medicine, Creighton University School of Medicine, Omaha, NE, USA; ^2^Department of Pulmonary and Critical Care, Creighton University School of Medicine, Omaha, NE, USA

## Abstract

**Background:**

Right heart thrombus or clot in transit is a rare venous thromboembolism (VTE) with high mortality. COVID-19 infection has been associated with increased risk of such events. We present the case of a 63-year-old man with no traditional VTE risk factors who was diagnosed with a clot in transit three weeks after diagnosis of COVID-19. *Clinical Case*. A 63-year-old male with no significant past medical history who presented to the emergency department with shortness of breath. He tested positive for COVID-19 three weeks prior. His oxygen saturation was 60% on room air and was put on nonrebreather mask. He was still showing signs of respiratory distress including tachypnea, tachycardia, diaphoresis, and accessory muscle use. The patient was subsequently intubated and mechanically ventilated. Chest computed tomography with contrast showed acute bilateral pulmonary emboli with flattening of the interventricular septum suggestive of right heart strain. Bedside echocardiogram showed severely enlarged right ventricle with reduced systolic function and evidence of right ventricular strain and a mobile echodensity in the right ventricle attached to the tricuspid valve consistent with a clot in transit. The patient was treated with full dose systemic thrombolysis with rapid improvement in his symptoms. He was extubated the following day and a repeat echocardiogram showed resolution of the clot in transit.

**Conclusion:**

Clot in transit is rare but can occur in COVID-19 patients even in the absence of traditional thromboembolism risk factors. Management includes systemic anticoagulation, systemic thrombolysis, and surgical embolectomy. Our patient was successfully treated with systemic thrombolysis.

## 1. Introduction

Right heart thrombus or clot in transit is a rare venous thromboembolism (VTE) with high mortality [1, 2]. COVID-19 infection has been associated with increased risk of such events. We present the case of a 63-year-old man with no traditional VTE risk factors who was diagnosed with a clot in transit three weeks after diagnosis of COVID-19.

## 2. Case Presentation

A 63-year-old male with no significant past medical history presented to the emergency department with shortness of breath. He tested positive for COVID-19 three weeks prior. He was initially asymptomatic but later developed a one-week history of shortness of breath on exertion and intermittent fevers. The patient's shortness of breath acutely worsened the night of presentation, so he called emergency medical services. His oxygen saturation was 60% on room air and he was ventilated with a bag-valve mask during transport.

**Figure 1:**
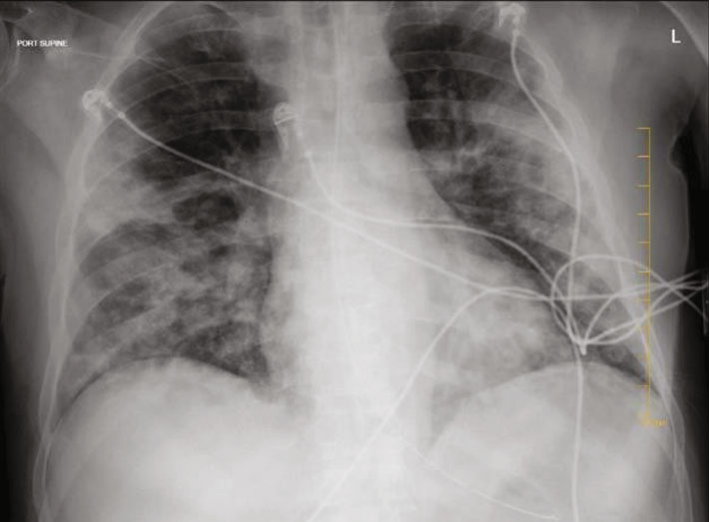
Chest X-ray showing bilateral lung opacities.

**Figure 2:**
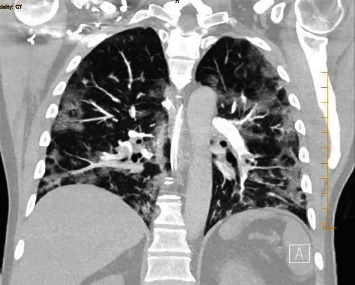
Chest computed tomography with contrast showing bilateral pulmonary emboli and multifocal lung consolidations.

**Figure 3:**
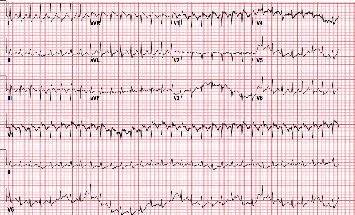
Electrocardiogram showing supraventricular tachycardia and the S1Q3T3 pattern (deep S waves in lead I and Q waves and T wave inversion in lead 3).

**Figure 4:**
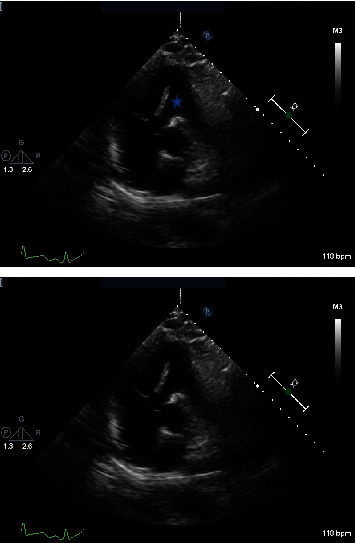
Echocardiogram in the short axis view showing the clot in transit in the right ventricle (star).

On arrival to the emergency department, the patient's vitals were heart rate 145/bpm, respiratory rate 34/minute, blood pressure 105/55 mmHg, temperature 36.6°C, and oxygen saturation 80%. Physical examination showed signs of respiratory distress including tachycardia, tachypnea, diaphoresis, and accessory muscle use. The patient was intubated and mechanically ventilated. Laboratory work-up was significant for sodium 126 mmol/l, creatinine 1.33 mg/dl, white blood cell count 18.9 k/*μ*l, lactic acid 7.8 mmol/l, troponin 0.08 ng/ml, and CHF peptide 350 pg/ml. Blood cultures were collected. Chest X-ray showed bilateral opacities consistent with multifocal pneumonia (Figure 1). Chest computed tomography with contrast (CTA) showed numerous acute segmental pulmonary emboli in the right upper, right lower, left upper, and left lower lobe pulmonary arteries as well as flattening of the intraventricular septum suggestive of right heart strain (Figure 2). The patient was started on a heparin drip and transferred to the intensive care unit for further management. Troponin was trended and peaked at 1.09 ng/ml. Repeat COVID-19 testing remained positive. The patient had an episode of supraventricular tachycardia that resolved after administration of adenosine. An EKG at the time showed supraventricular tachycardia as well as an S1Q3T3 pattern indicative of acute right heart strain (Figure 3). A transthoracic echocardiogram (TTE) was obtained and showed severely enlarged right ventricle with reduced systolic function and evidence of right ventricular strain, moderate tricuspid valve regurgitation, and a mobile echodensity in the right ventricle attached to the tricuspid valve consistent with a clot in transit (Figure 4). Lower extremity Doppler revealed an acute deep vein thrombosis of the left popliteal, posterior tibial, and peroneal veins. Vascular surgery was consulted. They determined that catheter-directed thrombolysis would be too high risk for this patient and instead administered a full dose of systemic alteplase with 100 mg over two hours for treatment. A heparin drip was then restarted. The patient's respiratory status improved quickly, and he was extubated the following day. A repeat TTE showed decreased right ventricular enlargement and no evidence of persistent thrombus. The patient continued to improve and was discharged on a direct-acting oral anticoagulant with outpatient pulmonology follow-up.

## 3. Discussion

A clot in transit describes a thrombus present in the right atrium or ventricle. It is rare with an incidence of 4% in patients with pulmonary embolism but has a mortality rate as high as 20 to 29% despite treatment [1–4]. Recently, several studies have shown an increased risk of thromboembolic events in patients with COVID-19 including pulmonary embolism (PE), deep vein thrombosis (DVT), ischemic stroke, and myocardial infarction. VTE was reported to occur in 18% of patients presenting to the emergency department with COVID-19 [5]. Patients hospitalized with COVID-19 were found to have a VTE rate of 3.4 to 11.9% among non-ICU patients [6, 7] and 13.6 to 69% among ICU patients [6, 8–10]. However, few case reports have been published describing COVID-19 patients developing clots in transit.

Although the exact mechanism by which coronavirus infection increases risk for VTE is not yet understood, there have been several proposed mechanisms including endothelial dysfunction, systemic inflammation, platelet activation, immobilization, hypoxia-induced activation of transcription factors, and the presence of lupus anticoagulant [8, 10–12]. The late presentation of VTE in the patient raises discussion about thromboembolic prophylaxis. Vechi et al. reported five cases of PE occurring three to four weeks after mild COVID-19 infection [13]. An autopsy study in France on out-of-hospital COVID-19 patients with unexplained death showed a higher rate of PE as the cause of death compared to a control group [14]. Despite the potential for increased VTE risk in COVID-19 infection, current CHEST guidelines do not recommend extended thromboprophylaxis due to insufficient data, although it can be considered in low bleeding-risk patients [15].

Our case also highlights the importance of utilizing TTE in cases of pulmonary embolism. The chest CTA showed segmental pulmonary emboli with evidence of right heart strain but did not reveal the clot in transit. TTE allowed for the detection of right heart thrombus which subsequently leads to appropriate management with systemic thrombolysis resulting in improvement of symptoms and resolution of the thrombus as evident by the repeat TTE. TTE is a bedside imaging modality that is widely available. It allows for the rapid detection of right heart thrombus treatment in a timely manner. This critical role of TTE has been recently demonstrated in several cases [16, 17].

No consensus for optimal treatment of clot in transit exists. Data is available from case series and meta-analyses, but no randomized controlled trials have been completed [2]. Potential options for treatment include systemic anticoagulation, systemic thrombolysis, and surgical embolectomy [2]. Several studies have shown survival benefit with systemic thrombolysis, but other studies have shown no mortality difference between treatment modalities [1, 3, 4]. Given the lack of clear evidence, the use of thrombolytic therapy for treatment of clot in transit should be based on an individualized approach assessing factors including clot size and location, bleeding risk, and risk of death. Contraindications to thrombolysis, including neurosurgery, head trauma, or stroke in the past 3 months, intracranial hemorrhage, blood pressure > 185/110 mmHg, active internal bleeding or bleeding diathesis, major surgery within the past 14 days, and known intracranial arteriovenous malformation, neoplasm, or aneurysm, must also be considered [18]. The decision to pursue systemic thrombolysis in our case was made by a multidisciplinary team based on our patient's high pulmonary embolism burden and critical illness.

## 4. Conclusion

The presented case describes a patient with no VTE risk factors who developed right heart thrombus three weeks after diagnosis of COVID-19 infection. The clot in transit was diagnosed with TTE and successfully treated with systemic thrombolysis. We highlight the importance of having a low threshold to suspect VTE in COVID-19 infection even in the absence of traditional risk factors, the role of TTE in diagnosis of right heart thrombus, and the therapeutic options in this cohort.

## Data Availability

No additional data was accessed or generated for this manuscript.

## References

[1] Islam M., Nesheim D., Acquah S. (2019). Right heart thrombi: patient outcomes by treatment modality and predictors of mortality: a pooled analysis. *Journal of Intensive Care Medicine*.

[2] Koć M., Kostrubiec M., Elikowski W. (2016). Outcome of patients with right heart thrombi: the Right Heart Thrombi European Registry. *The European Respiratory Journal*.

[3] Barrios D., Rosa-Salazar V., Morillo R. (2017). Prognostic significance of right heart thrombi in patients with acute symptomatic pulmonary embolism: systematic review and meta-analysis. *Chest*.

[4] Torbicki A., Galié N., Covezzoli A. (2003). Right heart thrombi in pulmonary embolism: results from the International Cooperative Pulmonary Embolism Registry. *Journal of the American College of Cardiology*.

[5] Gervaise A., Bouzad C., Peroux E., Helissey C. (2020). Acute pulmonary embolism in non-hospitalized COVID-19 patients referred to CTPA by emergency department. *European Radiology*.

[6] Bilaloglu S., Aphinyanaphongs Y., Jones S., Iturrate E., Hochman J., Berger J. S. (2020). Thrombosis in hospitalized patients with COVID-19 in a New York City health system. *Journal of the American Medical Association*.

[7] Santoliquido A., Porfidia A., Nesci A. (2020). Incidence of deep vein thrombosis among non-ICU patients hospitalized for COVID-19 despite pharmacological thromboprophylaxis. *Journal of Thrombosis and Haemostasis*.

[8] Klok F. A., Kruip M. J. H. A., Van der Meer N. J. M. (2020). Incidence of thrombotic complications in critically ill ICU patients with COVID-19. *Thrombosis Research*.

[9] Llitjos J., Leclerc M., Chochois C. (2020). High incidence of venous thromboembolic events in anticoagulated severe COVID-19 patients. *Journal of Thrombosis and Haemostasis*.

[10] Helms J., Tacquard C., Severac F. (2020). High risk of thrombosis in patients with severe SARS-CoV-2 infection: a multicenter prospective cohort study. *Intensive Care Medicine*.

[11] Bikdeli B., Madhavan M. V., Jimenez D. (2020). COVID-19 and thrombotic or thromboembolic disease: implications for prevention, antithrombotic therapy, and follow-up: JACC state-of-the-art review. *Journal of the American College of Cardiology*.

[12] Maatman T. K., Jalali F., Feizpour C. (2020). Routine venous thromboembolism prophylaxis may be inadequate in the hypercoagulable state of severe coronavirus disease 2019. *Critical Care Medicine*.

[13] Vechi H. T., Maia L. R., Alves M. D. M. (2020). Late acute pulmonary embolism after mild coronavirus disease 2019 (COVID-19): a case series. *Revista do Instituto de Medicina Tropical de São Paulo*.

[14] Benzakoun J., Hmeydia G., Delabarde T. (2020). Excess out-of-hospital deaths during COVID-19 outbreak: evidence of pulmonary embolism as a main determinant. *European Journal of Heart Failure*.

[15] Moores L. K., Tritschler T., Brosnahan S. (2020). Prevention, diagnosis, and treatment of VTE in patients with coronavirus disease 2019: CHEST guideline and expert panel report. *Chest*.

[16] Horowitz J., Yuriditsky E., Bakker J., Magda G., Teran F., Saric M. (2021). Clot in transit in a patient with COVID-19: transesophageal echocardiographic guidance of mechanical cardiopulmonary resuscitation. *CASE: Cardiovascular Imaging Case Reports*.

[17] Horowitz J. M., Yuriditsky E., Henderson I. J., Stachel M. W., Kwok B., Saric M. (2020). Clot in transit on transesophageal echocardiography in a prone patient with COVID-19 acute respiratory distress syndrome. *CASE Cardiovasc Imaging Case Reports.*.

[18] Baig M. U., Bodle J. (2022). Thrombolytic therapy. https://www.ncbi.nlm.nih.gov/books/nbk557411.

